# Binucleation of male accessory gland cells in the common bed bug *Cimex lectularius*

**DOI:** 10.1038/s41598-019-42844-0

**Published:** 2019-04-24

**Authors:** Koji Takeda, Jun Yamauchi, Aoi Miki, Daeyun Kim, Xin-Yeng Leong, Stephen L. Doggett, Chow-Yang Lee, Takashi Adachi-Yamada

**Affiliations:** 1 Department of Life Science, Faculty of Science, Gakushuin University, 1-5-1 Mejiro, Toshima-ku, 171-8588 Tokyo, Japan; 2 Urban Entomology Laboratory, Vector Control Research Unit, School of Biological Sciences, Universiti Sains Malaysia, 11800 Penang, Malaysia; 3 Present Address: Department of Entomology, Faculty of Agriculture, Kasetsart University, Bangkok, 10900 Thailand; 4 Centre for Chemical Biology, Universiti Sains Malaysia, 10 Persiaran Bukit Jambul, 11900 Penang, Malaysia; 5 Department of Medical Entomology, NSW Health Pathology, Westmead Hospital, Westmead, NSW 2145 Australia

**Keywords:** Nucleus, Evolutionary developmental biology

## Abstract

The insect male accessory gland (MAG) is an internal reproductive organ responsible for the synthesis and secretion of seminal fluid components, which play a pivotal role in the male reproductive strategy. In many species of insects, the effective ejaculation of the MAG products is essential for male reproduction. For this purpose, the fruit fly *Drosophila* has evolved binucleation in the MAG cells, which causes high plasticity of the glandular epithelium, leading to an increase in the volume of seminal fluid that is ejaculated. However, such a binucleation strategy has only been sporadically observed in Dipteran insects, including fruit flies. Here, we report the discovery of binucleation in the MAG of the common bed bug, *Cimex lectularius*, which belongs to hemimetabolous Hemiptera phylogenetically distant from holometabolous Diptera. In *Cimex*, the cell morphology and timing of synchrony during binucleation are quite different from those of *Drosophila*. Additionally, in *Drosophila*, the position of the two nuclei in the adult stage changes as a result of the mating history or the nutrient conditions; however, it remains stable in *Cimex*. These differences suggest that binucleation in the *Cimex* MAG plays a unique role in the male reproductive system that is distinct from that of *Drosophila*.

## Introduction

In addition to sperm, insect seminal fluid contains various components, such as proteins, peptides, amino acids, inorganic salts, and nitrogen-containing compounds. All of these components are produced and secreted from the male reproductive tract, which includes the ejaculatory duct^1^, ejaculatory bulb^2^, and the male accessory gland (MAG), the latter of which is an internal reproductive organ that is analogous to the mammalian prostate^3^. Various observations suggest that the size of the MAG is more important for male reproductive success than the size of the testes. For example, the MAGs of a variety of insects such as bush crickets (Orthoptera: Tettigoniidae), dobsonflies (Megaloptera: Corydalidae), and fireflies (Coleoptera: Lampyridae) produce spermatophores, which are protein capsules containing sperm and nutrients that are passed from males to females as a nuptial gift during mating^4–7^. Thus, larger spermatophores tend to assist the females in producing more eggs. In butterflies, larger spermatophores inhibit the access of spermatophores from other males^8,9^. In a genetically amenable species of fruit fly, *Drosophila melanogaster*, the functions of certain accessory gland proteins (Acps) have been extensively studied. One of these proteins, Sex Peptide (SP, also known as Acp70A), acts after it is transferred into the female bursa copulatrix. SP controls the behavior of the female and causes it to reject further mating with other males to reduce the risk of sperm competition^10,11^. SP is also known to bind to sperm in female storage organs^12^. Thus, males with larger MAGs, as well as those with a larger volume of sperm, have an increased probability of producing offspring by blocking other males^13,14^. In *Drosophila*, all of the epithelial cells of the MAG display a unique binucleation structure, which synchronously forms at the mid-pupal stage by skipping cytokinesis^15^. This binucleation is believed to serve a purpose in augmenting the morphological plasticity in the epithelium to provide a transition between the cuboidal and squamous cells, which in turn results in larger volumes of seminal fluids being sent to the females^16,17^. However, such binucleation in the MAGs has not been reported in other taxa of insects except for two of the species of Tephritid fruit flies, *Ceratitis capitata*^18^ and *Bactrocera tryoni*^19^.

In comparison, there has been only one report in which the MAG cells in a Hemipteran genus, *Dysdercus*, were observed to have single or multiple nuclei^20^. This phenomenon appears to be unique to this taxon and is not common to other taxa within the Hemiptera (TAY, unpublished observation). Through a microscopic survey of the MAG cells from various insect taxa, we found that the common bed bug, *Cimex lectularius*, has a MAG in which all epithelial cells are binucleated, similar to what was previously observed in *Drosophila*. However, in several aspects, the *Cimex* MAG cells display distinct differences from the *Drosophila* cells. In this report, we describe the cytological features of the *Cimex* MAG. The rarity of this binucleation in other insects would suggest that this feature evolved independently from *Drosophila* and that the role of binucleation in *Cimex* may be different from that of *Drosophila*.

## Results

### Binucleation of epithelial cells in the male accessory gland of *Cimex*

**Figure 1:**
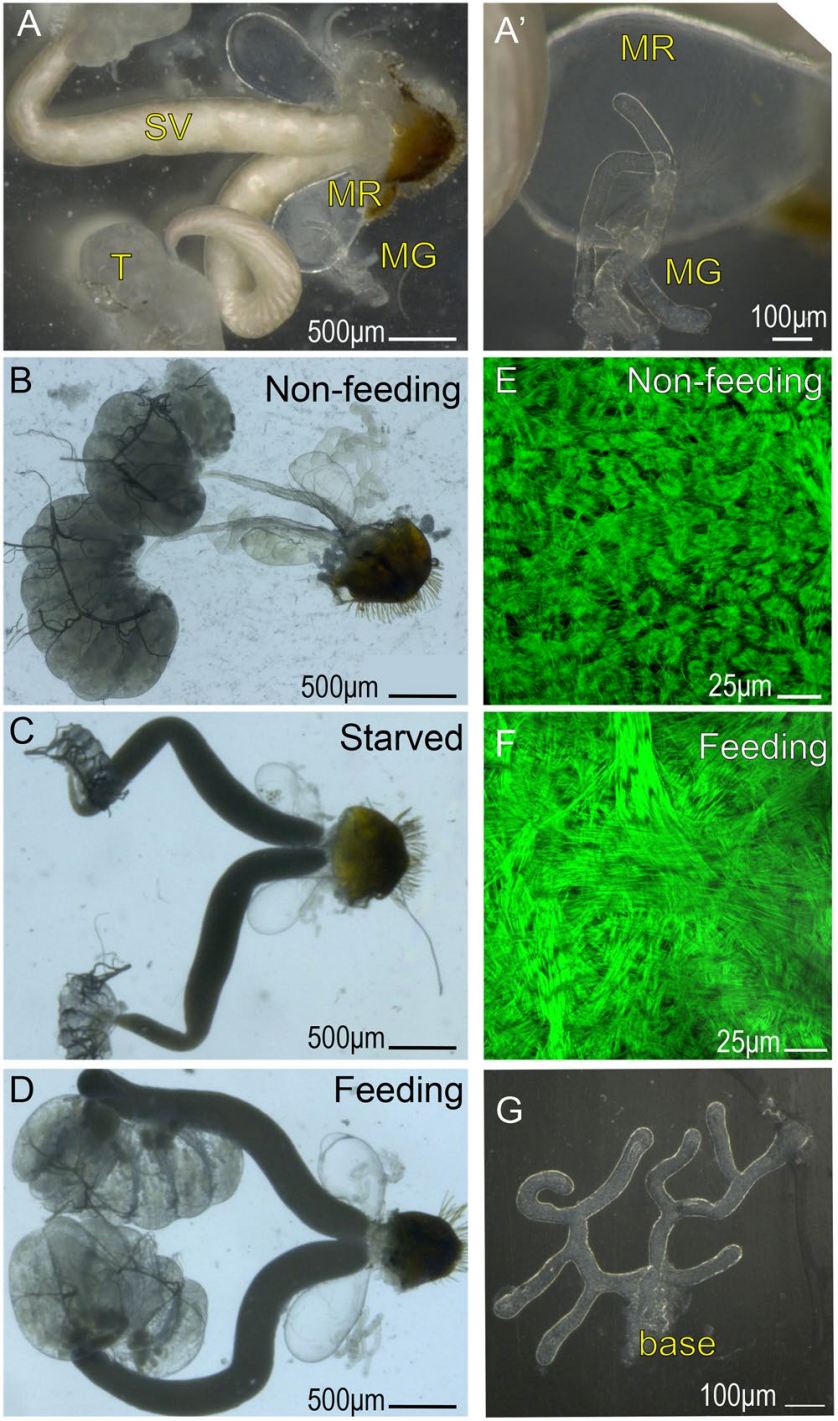
Growth of the internal reproductive system of *Cimex* males. (**A**) Male reproductive organs under epi-illumination. (**A’**) Enlargement of the MR and MG in A. (**B–D**) Male reproductive organs under transmitted light. (**B**) Under nonfeeding conditions (1 day after adult molting). (**C**) Under starved conditions (45 days after adult molting). (**D**) Under once a week feeding (more than 14 days after adult molting), the MRs grow in response to the nutrient intake, with less growth of the MG. Based on the comparison between B and C, feeding seems to be a more important factor than aging in the growth of the MR. (**E**,**F**) Outer muscle networks that are specifically found in the MR. (**G**) Development view of the MG. Several branch points are shown. Abbreviations: T, testis; SV, seminal vesicles; MR, mesadenial reservoir; MG, mesadenial gland. Some testicular follicles are collapsed in B.

In most insects, the MAG has a tubular sac-like morphology that is formed by a monolayer of epithelial cells that produce and secrete the various proteinous components of the seminal fluid (see Supplementary Fig. S2A for the fruit fly, *Drosophila*). The internal cavity of the MAG works as a reservoir for these substances, and during mating, it is squeezed by the many muscles that surround the celomic surface of the MAG. For *Cimex*, the morphology of the MAG is composed of two different parts, the mesadenial gland (MG) and the mesadenial reservoir (MR), both of which are connected at the base of the MG^21^ (Fig. 1A’,G). The MG is considered to be responsible for the production and secretion of various seminal fluid components, while the MR is responsible for the storage and evacuation of the MG products, which are thought to flow naturally from the MG to MR. The MG in *Cimex* resembles the MAG of other insects in its tubular shape; however, in *Cimex*, the MAG has several branches and lacks the circular muscles on the celomic surface of the epithelium. Furthermore, the MR has a unique omnidirectional muscular network, presumably for ejecting the MG products (Fig. 1E,F). Therefore, the production and storage of seminal fluids occur in different locations in *Cimex*, while they occur in the same location in other insects. The testes, seminal vesicles, and MRs in the adult males grow in response to the intake of nutrients a few weeks after molting to the adult stage, although the growth rates of these organs are different (Fig. 1A–D)^21^. Although the role of each *Cimex* MAG protein has not yet been examined, they are thought to be important to male reproductive success, as is the case for other insects. The volume of seminal fluid that is maintained is known to be important in determining male mating ability^21^.

**Figure 2:**
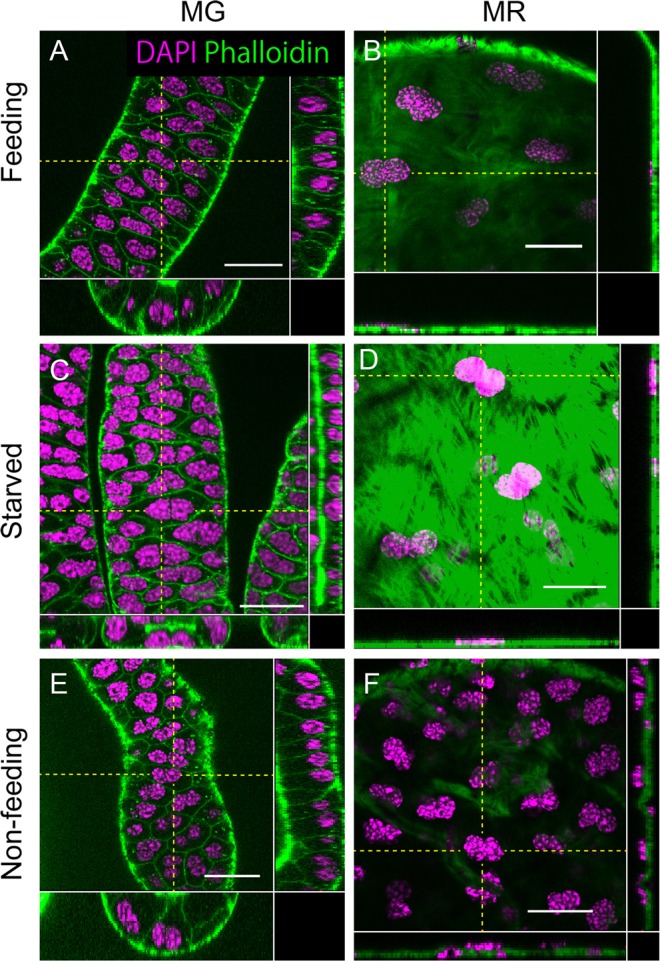
Epithelial cells in the mesadenial gland/reservoir are binucleated, and their nuclear position relative to the epithelial plane is unaffected by nutrient conditions. (**A,C,E**) MG. (**B,D,F**) MR. (**A** and **B**) Under feeding once a week for more than two weeks. (**C,D**) After starvation for 1 month. (**E,F**) Under nonfeeding conditions (1 day after adult molting). (Square photos in **A–F**) Surface views. (Vertical rectangular photos in **A–F**) Longitudinal sectional views that have been reconstituted from the Z-stacks of the confocal images on the yellow dashed vertical lines in the square photos in (**A–F**). (Horizontal rectangular photos in **A–F**) Transverse sectional views that have been reconstituted from the Z-stacks of the confocal images on the yellow dashed horizontal lines in the square photos in (**A–F**). Green: phalloidin staining. Magenta: DAPI staining. Scale bars in (**A–F**) are 25 μm. Although the epithelial cells along the lateral edge of the MG frequently appear to be in the mononucleate state at a glance, the two nuclei of these cells overlap at this angle of view. Note that the F-actin staining on the plasma membrane by phalloidin in the MRs is not visible because of the strong outer muscle staining. The growth in the size of the cells in the MR can be noted from the distance between the nuclear sets.

**Figure 3:**
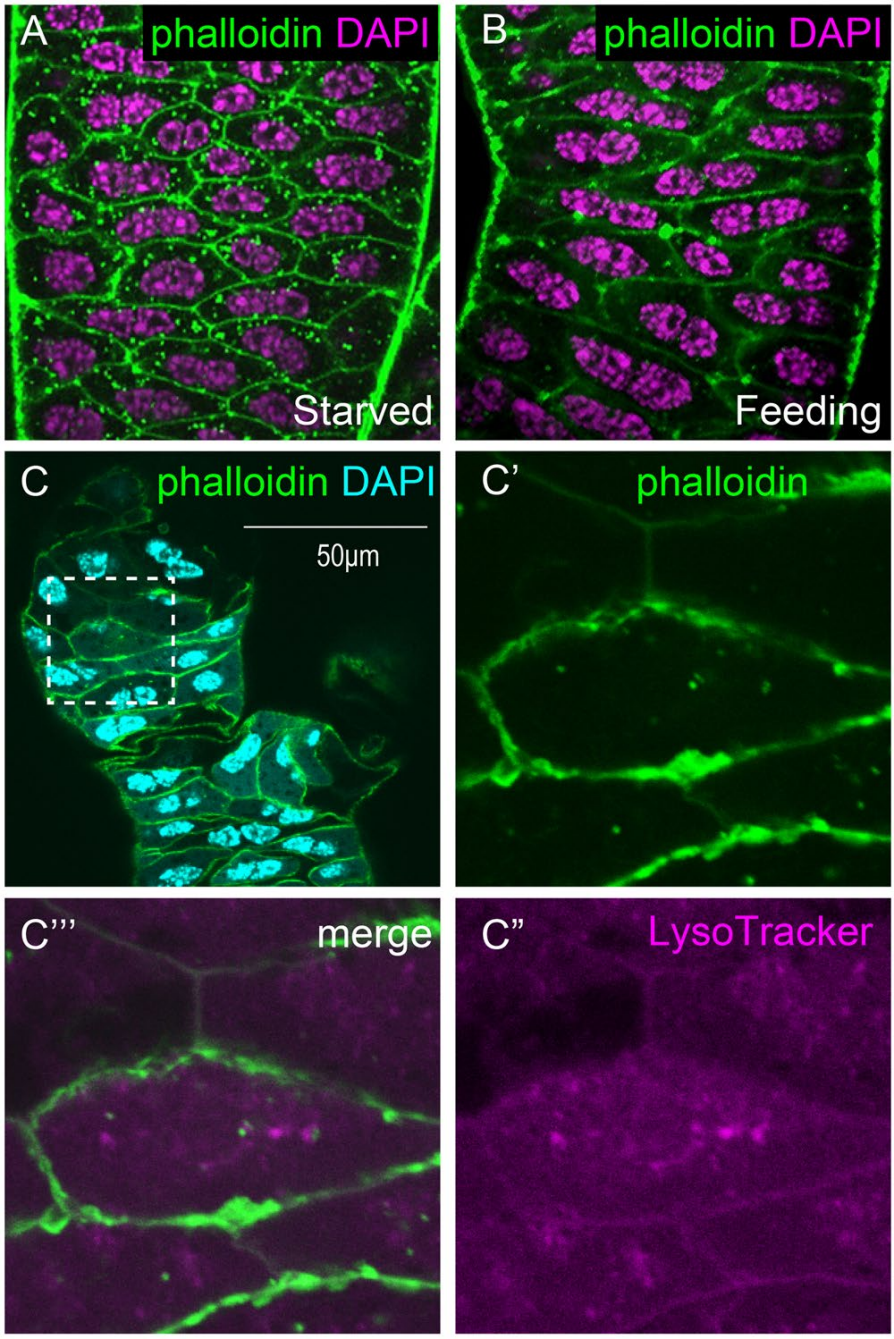
Autophagy in the mesadenial gland cells under starved conditions. (**A,B**) Difference in the amount of phalloidin-positive puncta in the MG epithelial cells between the starved (**A**) and feeding (**B**) conditions. Green: phalloidin staining. Magenta: DAPI staining. (**C**–**C”’**) After 1 month of starvation, the MG was double-stained with phalloidin (green) and LysoTracker (magenta). (**C**) Low magnification view of the double-stained MG. (**C’**–**C”’**) Enlargement of the boxed area in C. (**C’**) Phalloidin channel. (**C”)** LysoTracker channel. (**C”’**) Merged image. Green: phalloidin staining. Magenta: LysoTracker staining. Cyan: DAPI staining.

With simultaneous staining of the nuclei and actin filaments on the plasma membrane, binucleation of the epithelial cells was observed in both the MG (Fig. 2A,C,E) and the MR (Fig. 2B,D,F) of *Cimex*. A related species, the tropical bed bug *Cimex hemipterus*, also shows a similar binucleation pattern in the MAG (KT and TAY, unpublished observation). In the case of *Drosophila*, the cross-sectional area of the MAG cells and the apicobasal polarity of the two nuclei positions are highly affected by the nutrient conditions^16^ (see also Supplementary Fig. S1). Furthermore, both nymph and adult *Cimex* have a lifestyle that alternates between starvation and satiation through their intermittent sucking of blood from mammals. Therefore, we tried to compare the cell morphologies of the *Cimex* MAG between both of these life stage conditions. The growth of each reproductive organ, as shown in Fig. 1, did not affect the binucleation frequency (Fig. 2A–F), although the levels of nutrient-dependent growth in cell size could be recognized. The number of phalloidin-positive puncta increased in the cytoplasm of each MG cell under the starved condition (Fig. 3A,B), and these are considered to be parts of the autophagosomes that are formed during starvation-induced autophagy. It has also been reported that the early step of autophagy displays an accumulation of filamentous actin (F-actin), which is a target of phalloidin and participates in the sphere-like membrane vesicle formation for autophagosomes^22^. The autophagosomes later fuse with lysosomes for the degradation of their internal materials^23^. In fact, the phalloidin-positive puncta in the *Cimex* MAG cells are often overlapped or juxtaposed with the puncta stained by LysoTracker, which is a marker for acidic cytoplasmic membrane vesicles such as lysosomes (Fig. 3C–C”’). Thus, the phalloidin-positive puncta should be interpreted as signs of autophagy and can therefore be used to monitor the effect of starvation.

### Stable nuclear orientation of the apicobasal cell polarity irrespective of nutrient conditions in *Cimex*

In *D. melanogaster*, the position of the two nuclei along the apicobasal polarity of each epithelial cell in the MAG easily changes in response to nutrient conditions or mating experience^16^. When the MAG is expanded by secretion products under a high nutrient condition or before mating, the two nuclei are horizontally located on the epithelial plane to form squamous cells to maximize the storage of the secretion products. In contrast, when the MAG is shrunken under low nutrient conditions or after mating, the two nuclei are located angularly in reference to the epithelial plane, thus forming cuboidal cells, which minimize the internal volume^16,17^. In the related species *D. pseudoobscura*, this feature appears more exaggerated and shows a dual mode of the two contrasting cell morphologies of squamous and columnar cells (Supplementary Fig. S1).

We thus examined the position of the two nuclei along the apicobasal polarity in the *Cimex* MAG cells by observing cross-sectional images under various conditions of nutrient intake. Consequently, the apicobasal positioning of the two nuclei in most of the cells was stably horizontal to the epithelial plane under nonfeeding, starved, and feeding conditions both in the MG (Fig. 2A,C,E) and in the MR (Fig. 2B,D,F). These results suggest that the binucleation in the *Cimex* MAG does not contribute to the interconversion of cell shape between squamous and cuboidal (columnar) cells, as observed in *Drosophila*, and this binucleation may have some unknown roles in MAG function or development.

### Occurrence of binucleation just prior to adult molting

**Figure 4:**
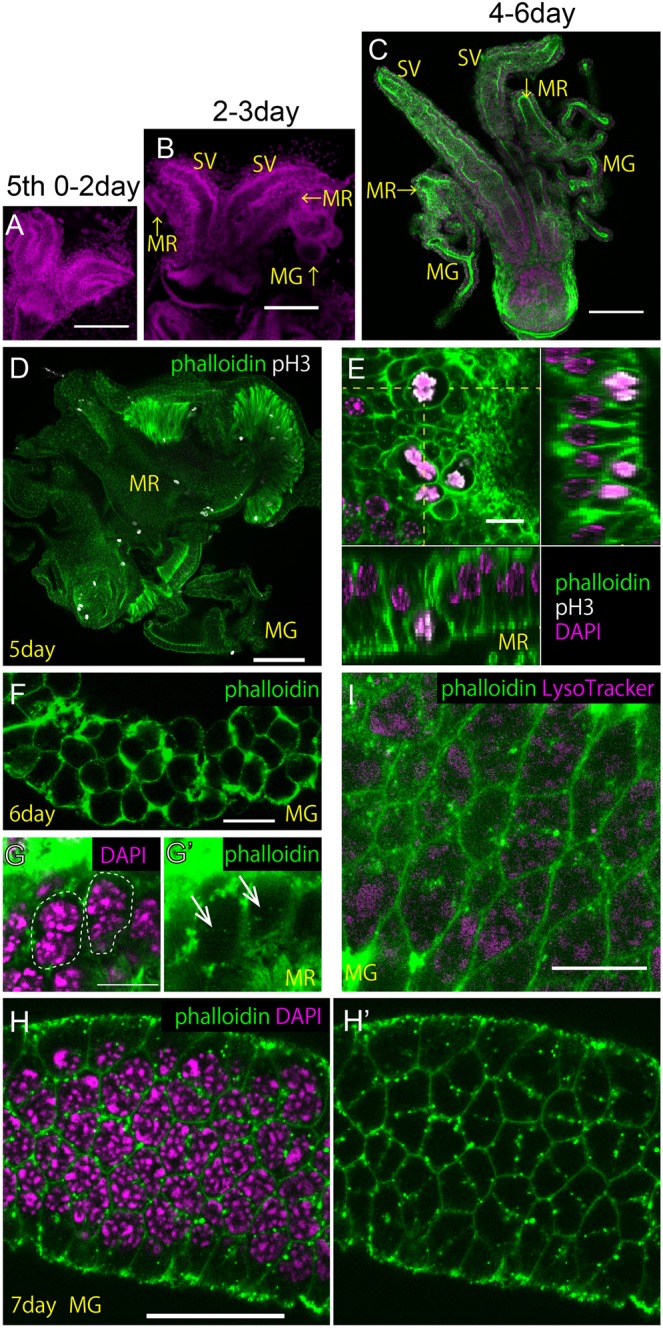
The binucleation process was observed in the late 5th instar nymph of *Cimex*. (**A–C**) Growth of internal reproductive organ primordia during the 5th instar nymph stage. At the (**A**) 0–2nd, (**B**) 2nd-3rd, and (**C**) 4th-6th days after molting to the 5th instar nymph. SV: seminal vesicle. MR: mesadenial reservoir. MG: mesadenial gland. Green: phalloidin staining. Magenta: DAPI staining. Scale bars in A-C are 100 μm. (**D**,**E**) Cells in the last mitotic phase were detected by staining with the anti-pH3 antibody (white) at the 5th day after molting to the 5th instar nymph. (**D**) Low magnification surface view of the MG and MR. (**E**) High magnification views of the MR. (Square photo in **E**) Surface view. (Rectangular photos in **E**) Longitudinal and transverse sectional views that were reconstituted from the Z-stacks of the confocal images on the yellow dashed lines in the square photo in E. Green: phalloidin staining. White: pH3 staining. Magenta: DAPI staining. Scale bars in (**D**,**E)** are 100 and 10 μm, respectively. Note the spherical shapes of the mitotic phase cells on the apical surface of the MR epithelium and the polarities of nuclear division horizontal to the epithelial plane. (**F**) MG cells after their last cytokinesis at the 6th day after molting to the 5th instar nymph. (**G**–**H’**) Cells during binucleation at the 7th day after molting to the 5th instar nymph. In the MR (**G**,**G’**), arrows indicate the phalloidin-positive puncta between the two nuclei. Dashed lines denote the outlines of the two epithelial cells. In the MG (**H**,**H’**), most of the newly binucleated cells display linear arrays of phalloidin-positive puncta between the two nuclei. Green: phalloidin staining. Magenta: DAPI staining. Scale bars in G and H are 10 and 25 μm, respectively. (**I**) Double staining by phalloidin (green) and LysoTracker (magenta) in the MG during binucleation. The LysoTracker-positive puncta are rarely observed and are not associated with phalloidin-positive puncta, unlike those of the starvation-induced autophagy (Fig. 3). The scale bar in I is 10 μm.

During the *Drosophila* pupal stage, the developing MAG shows a synchronous binucleation that results from skipping cytokinesis^15^. However, *Cimex* is a hemimetabolous insect without a pupal stage. Therefore, one question is how binucleation occurs in the *Cimex* MAG primordia during development. We dissected the internal reproductive organs or the primordia during the period from the 5th instar nymph to the adult stage and stained the organs with an anti-phospho-histone H3 (pH3) antibody to detect the mitotic phase cells in the developing MAG. The pH3 protein is known to appear specifically in the mitotic phase in a broad range of animal cells through its phosphorylation by some serine/threonine kinases such as Aurora B^15,17^. After molting into the adult, no cells were pH3-positive. In the late 5th instar nymph, tiny developing epithelial sacs can be observed, and these are considered to be primordia of the MR, based on their position and branching from the base of the seminal vesicles (Fig. 4A–C). Furthermore, a small protrusion with fewer branches was found on each MR primordium that was thought to be a prospective MG (Fig. 4B). In these MAG primordia, we observed scattered cells in the mitotic phase (Fig. 4D) that appeared to be similar to the binucleation of the *Drosophila* pupal MAG primordia^15^, although the levels of synchronization of the mitotic phase were much lower than those in *Drosophila*. Since pH3-positive staining was no longer observed after this stage, it was predicted that the cells would not divide further and would execute binucleation after the last mitotic phase. Such a terminal differentiation of the reproductive organs at the timing of adult development is considered reasonable because it inhibits the premature development of nymphs under low nutrient conditions.

**Figure 5:**
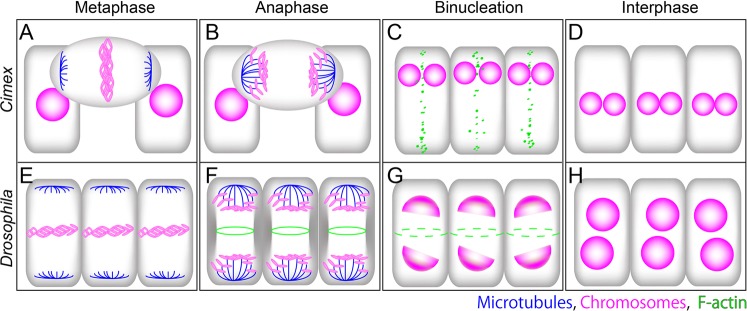
Comparison of the cell morphology and development during binucleation between *Cimex* and *Drosophila*. (**A–D**) *Cimex*. (**E–H**) *Drosophila*. (**A,E**) Metaphase. (**B,F**) Anaphase. (**C,G**) Binucleation. The contractile rings disappear before the completion of cytokinesis in *Drosophila*, while the partitions between the adjacent cells disappear after cytokinesis in *Cimex*. (**D,H**) Interphase. Blue: microtubules. Magenta: nuclei. Green: F-actin.

Generally, mitotic cells in a monolayer epithelium show delamination, detach from the basement membrane to form spherical cell shapes on the apical surface of the epithelium, and then display a horizontal spindle polarity relative to the epithelial plane. However, in the binucleation of the *Drosophila* MAG, the mitotic cells do not show delamination to retain their apicobasal polarity in the epithelium and display a spindle polarity that is vertical to the epithelial plane (Fig. 5E,F)^15^. We thus observed the detailed morphology of the cells around this binucleation stage in *Cimex*. Similar to cells that are undergoing standard mitosis, the cells in the binucleation stage became detached from the basement membrane and exhibited spherical shapes on the apical surface and a horizontal karyokinesis polarity relative to the epithelial plane in anaphase (Fig. 4E). For *Drosophila* MAG binucleation, after this stage, we observed telophase to be associated with a weak constriction of the mid-zone in the plasma membrane by the F-actin-accumulated contractile ring, which then breaks down after a short period. In *Cimex*, however, we never found such a vestigial contractile ring; instead, the F-actin-accumulated linear array of puncta between the two nuclei appeared synchronously in most of the cells (Fig. 4G,G’,H,H’). This structure appeared to disappear after a short period, which reminded us of the autophagy process shown in Fig. 3. However, because we did not detect any simultaneous staining by LysoTracker (Fig. 4I), the F-actin-positive puncta were not regarded as autophagosomes and were thought to be part of a fragmented plasma membrane that had been degraded by an unknown mechanism. Together with these findings, we concluded that the binucleation of *Cimex* MAG cells is achieved through the removal of the partition between two adjacent cells (Fig. 4F), which is quite different from what occurs in *Drosophila* (Fig. 5).

### Relationship between the nuclear position and proximodistal polarity

**Figure 6:**
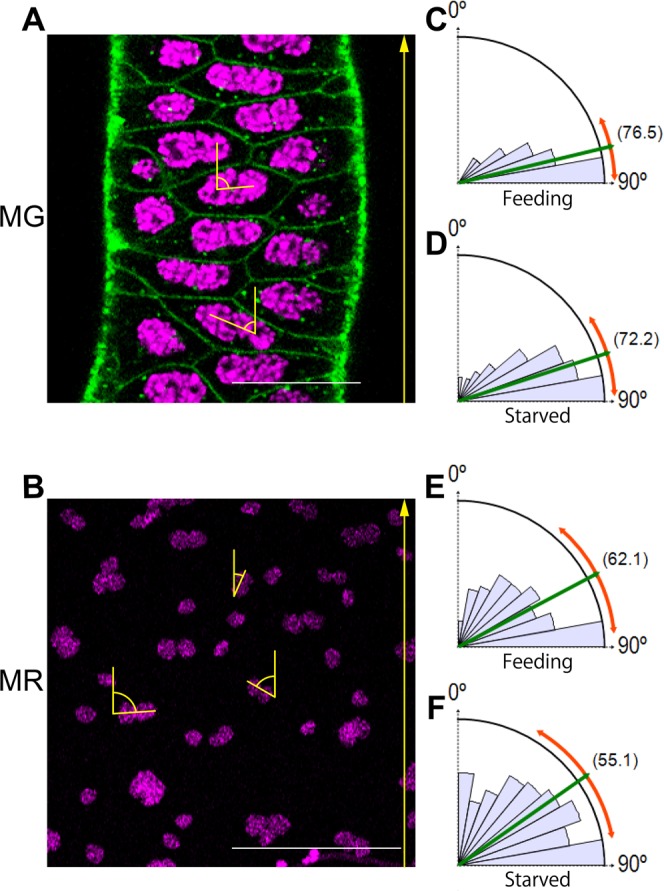
In *Cimex*, the angle of the line connecting the two nuclei relative to the proximodistal axis is stable in the mesadenial gland but variable in the mesadenial reservoir. (**A,C,D**) MG under feeding conditions. (**B,E,F**) MR under feeding conditions. (**A,B**) Representative pairs of nuclei with angles of the lines connecting the two nuclei to the proximodistal axis (yellow arrows from proximal to distal) in the MG and MR. Since the small nuclei that are shown to be in a scattered distribution in the MR are in the muscle cells but not in the epithelial cells, they were excluded in the angle measurement. Green: phalloidin staining. Magenta: DAPI staining. Scale bars in A and B are 25 and 100 μm, respectively. (**C–F**) Rose graphs showing the frequencies of each angle range. (**C,E**) Under feeding conditions. (**D,F**) Under starved conditions. The averaged values (parenthesized) and standard deviations (outer red arc) of the angle of the line connecting the two nuclei relative to the proximodistal axis are 76.5 ± 12.0 (N = 179) in (**C)** and 72.2 ± 15.5 (N = 273) in (**D**). The P-value between (**C,D**) is 0.13. Similarly, the averages and standard deviations are 62.1 ± 22.5 (N = 163) in (**E**) and 55.1 ± 25.1 (N = 153) in (**F**). The P-value between (**E,F**) is 0.18.

As described above, binucleation in the *Cimex* MAG cells did not appear to provide a role for the higher plasticity of cell shape along the apicobasal polarity, as per *Drosophila*. By contrast, the existence of two nuclei horizontal to the epithelial plane led us to predict that two axes would arise in a planar cell polarity. One would be parallel to the line connecting two nuclei, while the other would be orthogonal to it. We call these axes the “parallel axis” and “orthogonal axis”, respectively. It is thought that cells cannot shrink along the parallel axes as much as they can along the orthogonal axes because of the restriction in spatial occupation by the two nuclei (Fig. 6A). Therefore, binucleated cells frequently appear as elliptical in shape or as an elongated polygon in their apical surface view (Fig. 2A,C,E). We examined the polarity traits in these horizontally elongated cells. When the angle of the line connecting the two nuclei to the proximodistal axis was measured in each cell, the MG and MR showed an apparent difference. In the MG, the angles of the lines connecting the two nuclei were stable orthogonally to the proximodistal axis in most cells (Fig. 6C,D), while in the MR (Fig. 6B), this feature appeared weakened and more random (Fig. 6E,F). In both organs, there were no statistically significant differences between the feeding (Fig. 6C,E) and the starved (Fig. 6D,F) conditions. For comparison, we further analyzed the MAGs of two *Drosophila* species under feeding (Supplementary Fig. S2). They showed an intermediate phenotype between those of the *Cimex* MG and MR. Specifically, the polarities connecting the two nuclei appeared to be random when viewed in wide areas but unidirectional in local areas. Thus, the two contrasting styles of cell polarity in the *Cimex* binucleated cells are novel findings that have not been observed in *Drosophila*.

## Discussion

### Evidence for the independent evolution of binucleation in the *Drosophila* MAG and the *Cimex* MG/MR

Hemiptera, which includes *Cimex*, is the second order after Diptera in which the binucleation of the MAG cells has been observed. The other taxa between these two orders in the insect phylogenetic tree have all shown standard mononucleated cells in the MAG, as far as we have examined (TAY, unpublished observation), suggesting the independent evolution of binucleation in these taxa. However, evolutionary distance alone is not sufficient to determine whether the binucleation events in both taxa are results of parallel evolution or convergence. Regarding this fact, developmental features such as the synchronized stages in binucleation, the cell morphology along the apicobasal polarity, and the cytokinesis behaviors just prior to binucleation provided useful information and were all different between the two taxa. Therefore, both binucleation events are not regarded as the outcome of an independent evolution of the same process but instead should be considered as arising from a convergence of different processes that resulted in a similar outcome.

### Possible roles for binucleation in the *Cimex* MG and MR

One of the unique morphological features in the *Cimex* MAG is that it is composed of two parts, the MG and the MR. Based on their relative position, size difference in response to nutrient intake, and the presence or absence of outer musculature, the MG and MR are predicted to have roles in production and storage, respectively. These functional differences may be related to the differences in polarity of the nuclear position, which is orthogonal to the proximodistal axis in the MG but more random in the MR. Specifically, the ordered array of cell like stacking bricks in the MG may provide structural stability to the tubular gland for the development of the directed branching and the appropriate extension of each branch in the MG (Fig. 1G). If some cells with different polarities of nuclear position are present in an ordered cellular array, the proper tubular morphology of the MG may be interrupted. In the MR, a random array in the polarities of the nuclear position may be suitable for the omnidirectional expansion into a reservoir for a maximal storage volume. Since a sphere has the same surface area and a larger volume, the tubular and directional growth may not be the most appropriate method for larger storage. As both orientations in the polarities of the nuclear position are not simultaneously compatible, the standard MAG that is found in most insects may compromise these two orientations in cell polarities in a single tubular and expandable MAG. In fact, the *Drosophila* MAG shows an intermediate type of polarity in its nuclear position, which is morphologically stable in local areas but is random in wide areas for maximal storage. This is the first proposal to describe the role of the planar cell polarity in binucleated cells in addition to the previously known role of their apicobasal polarities^15^.

## Methods

### Cimex and Drosophila

The *Cimex lectularius* that was used in this study is the Monheim strain, which is originally from Monheim (Bayer Laboratories) in Germany. This is an insecticide-susceptible strain that has been maintained in colony since the early 1970s and has not been exposed to insecticides^24^. The *Drosophila pseudoobscura* strain that was used in this study is the MV2-25 Baylor sequencing strain that was obtained from the Kyorin-fly stock center in Japan. The *Drosophila melanogaster* strain that was used in this study is the *w*^1118^ that was obtained from the Bloomington *Drosophila* stock center in the USA.

### Reagents and methods of MAG cell staining

The following reagents were used for tissue staining:

0.1 μg/ml of DAPI (4′,6-diamidino-2-phenylindole, Sigma) for nuclei staining.

1:100 dilution of Alexa Fluor 488-labeled phalloidin (Life Technol.) for F-actin staining.

1:100 dilution of rabbit anti-pH3 antibody (Upstate Biotech) for the staining of phospho-histone H3 on the chromosomes in mitotic phase.

1:200 dilution of mouse anti-Cora antibody (DSHB) for *Drosophila* Coracle staining.

1:200 dilution of Alexa Fluor 555-labeled anti-rabbit IgG (Jackson Immuno Research).

1:200 dilution of Alexa Fluor 555-labeled anti-mouse IgG (Jackson Immuno Research).

1:1000 dilution of LysoTracker Red DND-99 (Invitrogen) for lysosome staining. For fixed tissue staining, the method was performed as previously published^15^. For living tissue staining with both phalloidin and LysoTracker on the same specimen, the method was performed as follows. Tissues were dissected in phosphate buffered saline (PBS). The dissected tissues were soaked in PBS containing LysoTracker and left for 2 hours at 25 °C. After rinsing the excess LysoTracker with PBS, the tissues were fixed in 4% formalin for 20 minutes at 25 °C. After the removal of formalin with PBS rinsing, the tissues were stained with Alexa Fluor 488-labelled phalloidin overnight at 4 °C. After rinsing excess phalloidin with PBS, the tissues were observed with the FV3000 laser confocal microscope, as described below.

### Microscopic observation

Low magnification images of the entire reproductive system were obtained with a VHX-2000 digital microscope (Keyence). Laser confocal microscopic images of tissue sections were obtained with the Digital Eclipse C1Si (Nikon), FV1000 (Olympus), or FV3000 (Olympus).

### Angle measurement of the lines connecting the two nuclei in the MAG cells

In each of the *Cimex* MG and MR and the *Drosophila* MAG photographs, a curved midline was drawn to trace the proximodistal axis. The lines connecting the centroids of the two nuclei were also drawn in all of the examined binucleated cells. The angles of these lines to the proximodistal axis were measured and counted in 10-degree intervals by using the graphic software ImageJ (NIH, version 1.52a). The rose graphs were depicted by the graphic software Rose.NET 0.10.0.

### Statistical test

Differences in the angles of lines connecting two nuclei relative to the proximodistal axis between samples were statistically verified by Student’s t-test. Each value is described in the legend of Fig. 6.

## Supplementary information

Supplementary figures 1 and 2
