# Supplementary material for: Binucleation of male accessory gland cells in the common bed bug Cimex lectularius
Source: Sci Rep. 2019 Apr 24;9:6500. doi: 10.1038/s41598-019-42844-0 (PMC6482304; doi:10.1038/s41598-019-42844-0)
Supplement: Supplementary file 1 — Supplementary figures 1 and 2 [file 41598_2019_42844_MOESM1_ESM.pdf]

Supplementary information

Binucleation of male accessory gland cells in the common bed bug *Cimex lectularius*

Koji Takeda[1], Jun Yamauchi[1], Aoi Miki[1], Daeyun Kim[2,3], Xin-Yeng Leong[2,4], Stephen L. Doggett[5], Chow-Yang Lee[2], and Takashi Adachi-Yamada[1]

[1]Department of Life Science, Faculty of Science, Gakushuin University,

1-5-1 Mejiro, Toshima-ku, 171-8588 Tokyo, Japan.

[2]Urban Entomology Laboratory, Vector Control Research Unit, School of Biological Sciences, Universiti Sains Malaysia, 11800 Penang, Malaysia.

[3]Present address: Department of Entomology, Faculty of Agriculture, Kasetsart University, Bangkok 10900, Thailand.

[4]Centre for Chemical Biology, Universiti Sains Malaysia, 10 Persiaran Bukit Jambul, 11900 Penang, Malaysia.

[5]Department of Medical Entomology, NSW Health Pathology, Westmead Hospital, Westmead, NSW 2145, Australia.

Contact information: Takashi Adachi-Yamada,

Phone: +81-3-5902-9411, Fax: +81-3-5992-1029

E-mail address: Takashi.Adachi-Yamada@gakushuin.ac.jp

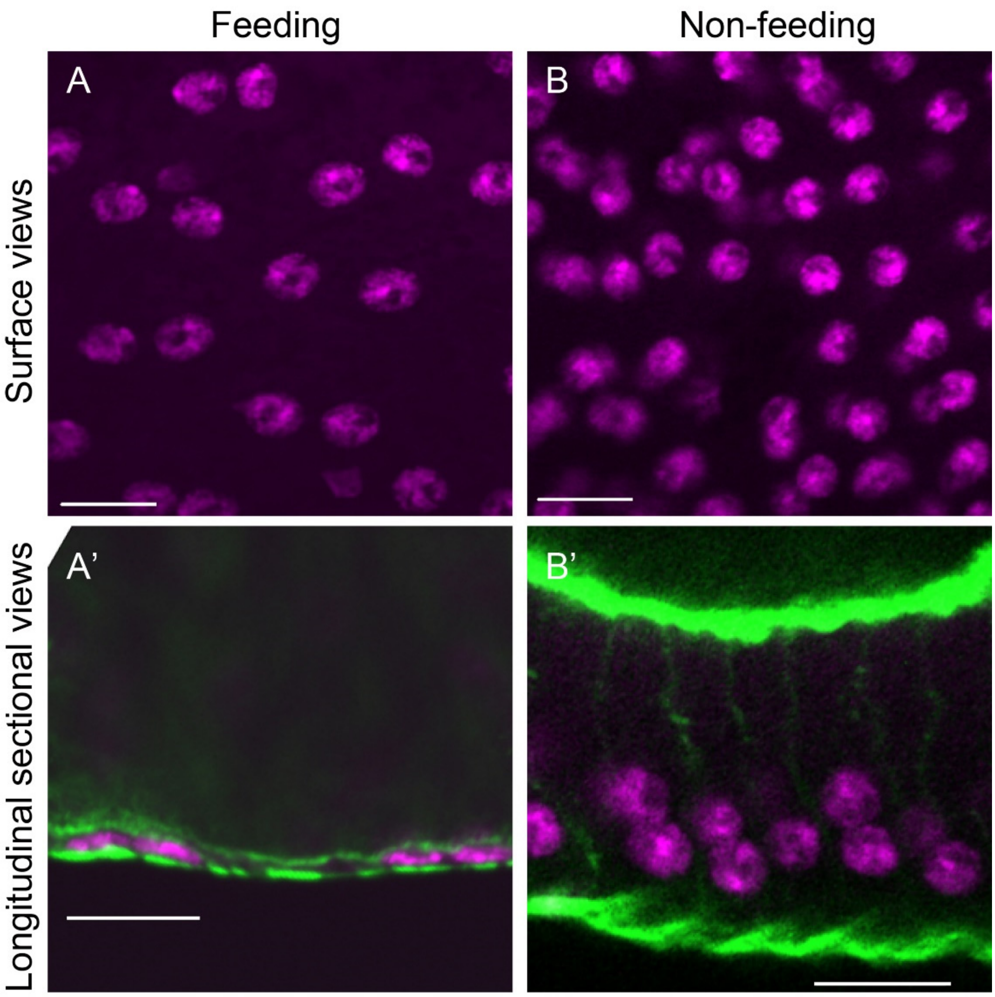

Supplementary Figure 1. Nuclear position relative to the epithelial plane in response to nutrient intake in the MAG of *Drosophila pseudoobscura*
(A and A') Under feeding conditions.
(B and B') Under nonfeeding conditions.
(A and B) Surface views.
(A' and B') Longitudinal sectional views.
Scale bars in A and B are 10 μm.

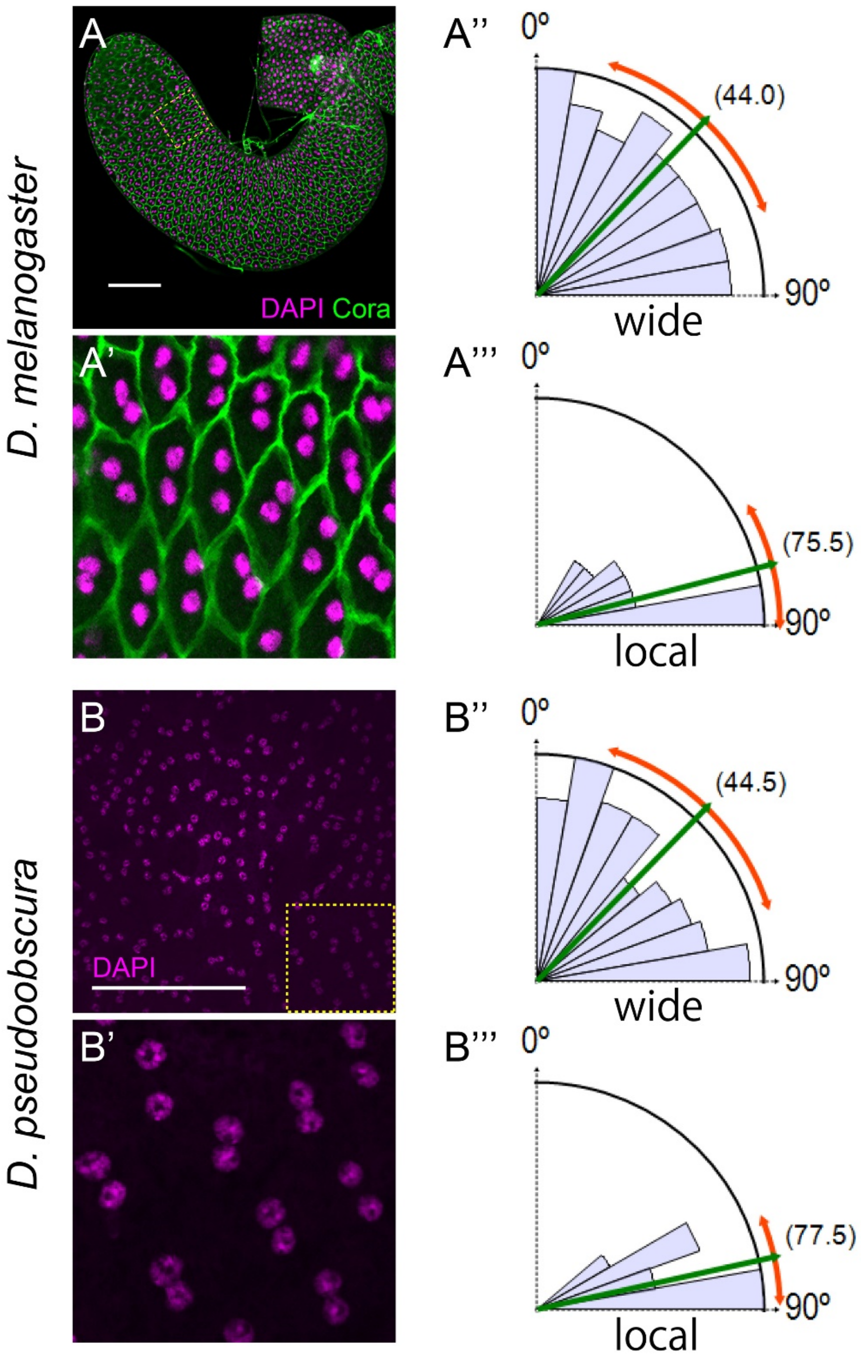

Supplementary Figure 2. In *Drosophila*, the angle of the line connecting the two nuclei relative to the proximodistal axis is variable in wide areas but stable in local areas in the MAG

(A and A') MAG of *Drosophila melanogaster* under feeding conditions.
(A'' and A''') Graphs showing the frequencies of each angle range.
(A and A'') Wide area. (A' and A''') Local area boxed in A.
(B and B') MAG of *Drosophila pseudoobscura* under feeding conditions.
(B'' and B''') Graphs showing the frequencies of each angle range.
(B and B'') Wide area. (B' and B''') Local area boxed in B.
Green: Cora staining (for plasma membrane). Since the anti-Cora antibody is raised against the Coracle protein from *D. melanogaster*, it does not stain this protein in *D. pseudoobscura*. Magenta: DAPI staining. The scale bars in A and B are 100 and 50 μm, respectively. The averaged values (parenthesized) and standard deviations (outer red arc) of the angle of the line connecting the two nuclei relative to the proximodistal axis are 44.0±26.7 (N=150) in A'' and 75.5±15.5 (N=18) in A'''. Similarly, the averages and standard deviations are 44.5±27.8 (N=139) in B'' and 77.5±11.0 (N=14) in B'''.
